# Calcium/calmodulin-dependent kinase IV contributes to translation-dependent early synaptic potentiation in the anterior cingulate cortex of adult mice

**DOI:** 10.1186/1756-6606-3-27

**Published:** 2010-09-16

**Authors:** Hiroki Toyoda, Ming-Gao Zhao, Valentina Mercaldo, Tao Chen, Giannina Descalzi, Satoshi Kida, Min Zhuo

**Affiliations:** 1 Department of Physiology, Faculty of Medicine, University of Toronto, Toronto, Ontario, Canada; 2 Department of Brain and Cognitive Sciences, College of Natural Sciences, Seoul National University, Seoul 151-746, Korea; 3 Department of Bioscience, Faculty of Applied Bioscience, Tokyo University of Agriculture, Tokyo, Japan; 4 Department of Neuroscience and Oral Physiology, Osaka University Graduate School of Dentistry, Suita, Japan; 5 Department of Pharmacology, Fourth Military Medical University, Xi'an, China

## Abstract

Calcium/calmodulin-dependent kinase IV (CaMKIV) phosphorylates the major transcription factor, cyclic AMP-responsive element binding protein (CREB), which plays key roles in synaptic plasticity and memory consolidation. Our previous study showed that long-term potentiation (LTP) in the anterior cingulate cortex (ACC) was significantly enhanced in transgenic mice overexpressing CaMKIV. Considering that the CaMKIV-CREB pathway plays a central role in the protein synthesis-dependent LTP, it is possible that upregulation of CaMKIV contributes to enhancement of LTP by promoting protein synthesis. To test this possibility, we examined the effects of transcription and translation inhibitors on synaptic potentiation induced by pairing of synaptic activity with postsynaptic depolarization (paired training) in ACC pyramidal neurons of wild-type and CaMKIV transgenic mice. We found that synaptic potentiation induced by paired training was partially inhibited by transcription or translation inhibitors both in wild-type and CaMKIV transgenic mice; the extent of inhibition was markedly larger in the CaMKIV transgenic mice than in the wild-type mice. Biochemical and immunohistochemical studies revealed that CaMKIV was distributed in the membrane, cytosol and nucleus of ACC neurons. Our results reveal in the first time a transcription- and translation-dependent component of early synaptic LTP in adult ACC synapses, and demonstrate that CaMKIV enhances early synaptic potentiation by activating new protein synthesis.

## Introduction

The long-lasting changes of neural circuitry in forebrain structures including the anterior cingulate cortex (ACC) are believed to contribute to emotion, learning, memory and pain [1-6], and such long-term changes in neural circuitry may require new protein synthesis. Long-term potentiation (LTP) is typically divided into early-phase and late-phase LTP, in which the latter is mainly thought to be dependent on protein synthesis. At the CA1 [7] and CA3 [8] synapses, protein synthesis inhibitors disrupt late-phase but not early-phase LTP. By contrast, other studies reported that early-phase LTP in CA1 [9], CA3 [10], and dentate gyrus [11] was suppressed by protein synthesis inhibitors (see Table 1). Thus, it is likely that protein synthesis-dependent mechanisms play critical roles in not only late-phase but also early-phase LTP, at least in part. However, little is known about whether transcription and translation affects early-phase LTP within ACC synapses.

**Table 1:** Effects of protein synthesis inhibitors on early-phase LTP (E-LTP) and late-phase LTP (L-LTP).

Brain region	E-LTP	L-LTP	References
CA1	Blocked		[9]
	Blocked		[28]
	No effect	blocked	[54]
	No effect	blocked	[8]
	No effect	blocked	[22]
	No effect	blocked	[55]
	Partially blocked	blocked	[29]
**CA3**	No effect	blocked	[56]
	Blocked		[30]
	Partially blocked		[10]
**Dentate gyrus**	No effect	blocked	[57]
	Partially blocked	blocked	[11]
**Spinal cord**	No effect	blocked	[58]
**Amygdala**	No effect	blocked	[59]
**Prefrontal cortex**	No effect	blocked	[60]
**ACC**	Partially blocked		This study

It has been well established that the cyclic AMP-responsive element binding protein (CREB) is a major transcription factor associated with long-term memory [12,13], and calcium-calmodulin-dependent protein kinase IV (CaMKIV) plays an essential role in activity-dependent CREB phosphorylation [14-16]. In the hippocampus, the CaMKIV-CREB pathway is required for protein synthesis-dependent late-phase LTP [17,18]. On the other hand, it is conceivable that CaMKIV is also involved in early-phase LTP, because our previous study has shown that early-phase LTP in the ACC, amygdala, insular cortex and somatosensory cortex was disrupted in CaMKIV knockout mice [19]. Additionally, we previously reported that early-phase LTP in ACC neurons of CaMKIV transgenic mice was significantly enhanced compared with those of wild-type mice [20]. Thus, it is possible that CaMKIV modulates early-phase LTP by regulating transcription and translation in ACC synapses. In our behavioral study, trace fear memory was significantly enhanced in CaMKIV transgenic mice, suggesting that CaMKIV affects the ability to sustain attention in a manner needed for retaining of the memory [20], although its mechanism remains unknown. Revealing how CaMKIV contributes to transcription and translation dependent-synaptic plasticity in ACC synapses will be helpful to understand ACC-related functions such as trace fear memory.

In the present study, we employed integrative approaches to investigate if the enhancement of early LTP by CaMKIV is dependent on transcription and translation. Here, we show that synaptic potentiation induced by paired training was significantly suppressed by transcription and translation inhibitors both in wild-type and CaMKIV transgenic mice; the extent of suppression of LTP was much larger in CaMKIV transgenic mice than in wild-type mice. Furthermore, biochemical and immunostaining observations revealed that CaMKIV is indeed distributed in the membrane, cytosol and nucleus of ACC neurons. These observations strongly suggest that overexpression of CaMKIV enhances early synaptic potentiation by promoting protein synthesis in ACC neurons.

## Results

### Effects of a transcription inhibitor on synaptic potentiation in ACC neurons

We have previously shown that synaptic potentiation in ACC neurons from the CaMKIV transgenic mice was significantly enhanced by spike-timing protocol which involves pairing three presynaptic stimuli, which caused three excitatory post-synaptic potentials (EPSPs) (10 ms ahead), with three postsynaptic action potentials at 30 Hz, paired 15 times every 5 s [20]. In the present study, we induced synaptic potentiation by pairing of synaptic activity with postsynaptic depolarization (or called paired training) (80 pulses of presynaptic stimulation at 2 Hz in layer V with postsynaptic depolarization at +30 mV) in the wild-type and CaMKIV transgenic mice. This protocol is useful to induce robust LTP in the ACC [4] and amygdala [21]. First, we investigated if actinomycin-D, a transcription inhibitor, affects synaptic potentiation in ACC neurons induced by the paired training. Actinomycin-D was used at a concentration of 40 μM, a dose that reduces uridine incorpolation into RNA by 77% [22]. We performed whole-cell patch-clamp recordings from visually identified pyramidal neurons in layer II/III of the ACC. Fast excitatory post-synaptic currents (EPSCs) were obtained by delivering focal electrical stimulation to layer V. We identified pyramidal neurons based on the pyramidal shape of their somata by putting Lucifer yellow into the intracellular solution and confirmed that the recordings were performed from cortical pyramidal cells by injecting depolarizing currents into the neuron [4].

**Figure 1:**
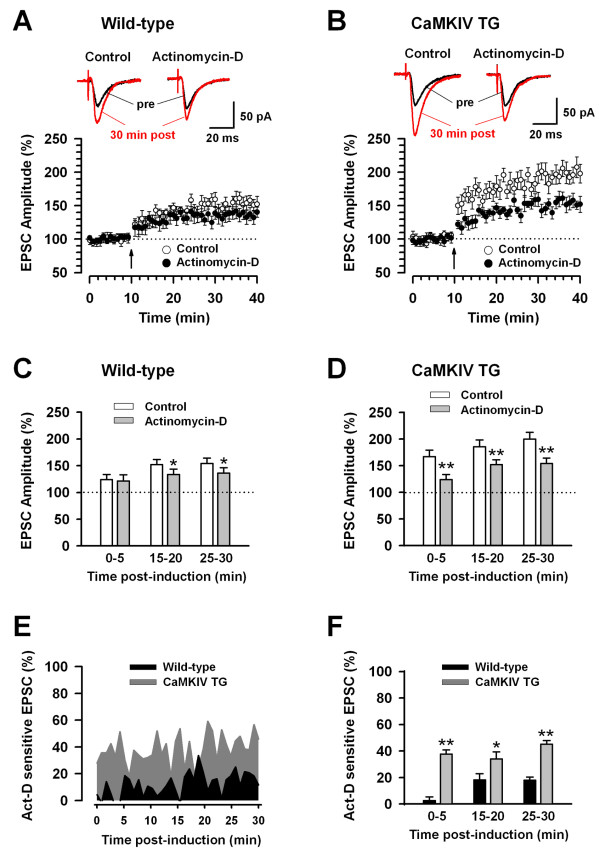
**Enhanced synaptic potentiation in ACC neurons of CaMKIV transgenic mice mediated by transcription activity**. A: The paired training inducing LTP in ACC neurons of wild-type mice (n = 8/5 mice). Partial inhibition of LTP by 40 μM actinomycin-D in ACC neurons of wild-type mice (n = 9/5 mice). B: Enhanced LTP in ACC neurons of CaMKIV transgenic (TG) mice (n = 8/5 mice). Marked inhibition of LTP by actinomycin-D in ACC neurons of CaMKIV TG mice (n = 7/5 mice). A-B: The insets showing averages of five EPSCs at baseline responses (pre) and at 25-30 min (30 min post) after the paired training (arrow); the dashed lines indicating the mean basal synaptic responses. C: Statistical results showing the significant inhibition of LTP by actinomycin-D at 15-20 and 25-30 min after induction of LTP in ACC neurons of wild-type mice. The dashed line indicating the mean basal synaptic responses. *P < 0.05 compared to control. D: Statistical results showing the significant inhibition of LTP by actinomycin-D at 0-5, 15-20 and 25-30 min after induction of LTP in ACC neurons of CaMKIV TG mice. The dashed line indicating the mean basal synaptic responses. **P < 0.01 compared to control. E: Actinomycin-D (Act-D) sensitive synaptic potentiation in ACC neurons of CaMKIV TG mice is larger than that of wild-type mice. F: Statistical results showing that Act-D sensitive synaptic potentiation in ACC neurons of CaMKIV TG mice is significantly larger than that of wild-type mice at 0-5, 15-20 and 25-30 min after induction of LTP. *P < 0.05, **P < 0.01 compared to wild-type mice.

The paired training induced a significant, long-lasting potentiation of synaptic responses in the ACC neurons of wild-type mice (154.4 ± 10.0%, n = 8/5 mice, *P *< 0.05 compared with baseline at 25-30 min post-induction, Fig. 1A and 1C). To investigate whether protein synthesis is involved in such synaptic potentiation, we tested the effect of a transcription inhibitor, actinomycin-D. In the ACC slices pretreated with 40 μM actinomycin-D for 30 min, LTP induced by the paired training was significantly suppressed compared with control (135.7 ± 9.6%, n = 9/5 mice, *P *< 0.05 compared with baseline at 25-30 min post-induction, Fig. 1A and 1C). Analysis of the time course of the effects of actinomycin-D on LTP in wild-type mice revealed that actinomycin-D significantly suppressed the synaptic potentiation at 15-20 and 25-30 min after LTP introduction (*P *< 0.05 compared with baseline, Fig. 1C), although there was no significant reduction at 0-5 min after introduction (Fig. 1C). This result suggests that paired training itself can drive protein synthesis by activating transcription at 15 min after LTP introduction in ACC neurons of wild-type mice.

In the CaMKIV transgenic mice, synaptic potentiation was significantly enhanced in comparison to the wild-type mice (199.7 ± 13.2% of baseline at 25-30 min post-induction, n = 8/5 mice, Fig. 1B and 1D, *P *< 0.05 compared with wild-type mice, see Fig. 1A and 1C), similar to the results obtained in our previous study [20]. When LTP was induced in the ACC slices of CaMKIV transgenic mice pretreated with actinomycin-D, the synaptic potentiation was remarkably suppressed (152.6 ± 9.7% of baseline at 25-30 min post-induction, n = 7/5 mice, *P *< 0.01 compared with control, Fig. 1B and 1D). The analysis of the time course of the effects of actinomycin-D on LTP in the CaMKIV transgenic mice revealed that actinomycin-D significantly suppressed the synaptic potentiation at 0-5, 15-20 and 25-30 min after LTP introduction (*P *< 0.01 compared with baseline, Fig. 1D). This result suggests that, as in wild-type mice, paired training itself can induce protein synthesis by activating transcription immediately after LTP introduction in ACC neurons of CaMKIV transgenic mice. By calculating the actinomycin-D sensitive synaptic potentiation in wild-type and CaMKIV transgenic mice, the effects of transcription on the synaptic potentiation caused by the paired training was compared between the two groups (Fig. 1E). The actinomycin-D sensitive synaptic potentiation in the ACC neurons of CaMKIV mice was significantly larger than that of wild-type mice at 0-5, 15-20 and 25-30 min after LTP introduction (*P *< 0.01 at 0-5 min post-induction, *P *< 0.05 at 15-20 min post-induction, *P *< 0.01 at 25-30 min post-induction, Fig. 1F). These results indicate that gene transcription was markedly enhanced in the ACC neurons of CaMKIV transgenic mice compared to those of wild-type mice.

### Effects of a translation inhibitor on synaptic potentiation in ACC neurons

**Figure 2:**
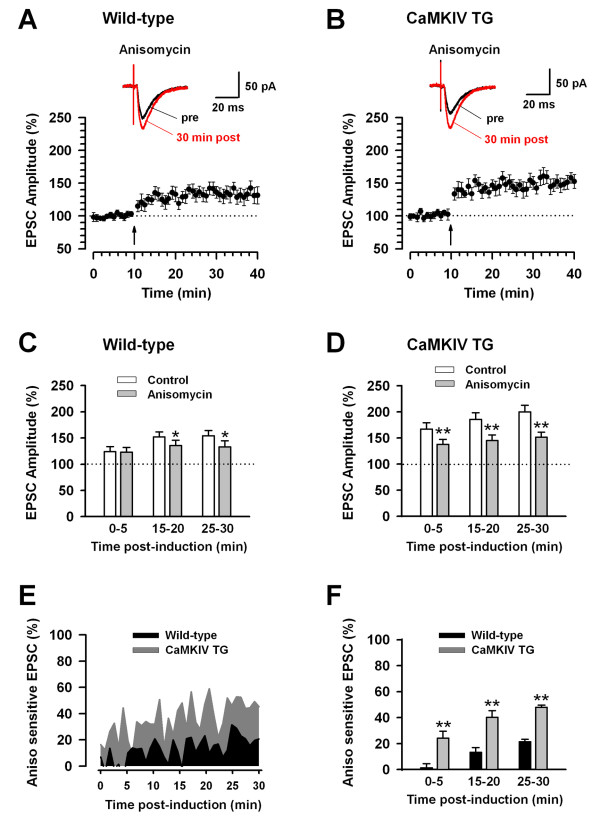
**Enhanced synaptic potentiation in the ACC neurons of CaMKIV transgenic mice mediated by translation activity**. A: Partial inhibition of LTP by 20 μM anisomycin in ACC neurons of wild-type mice (n = 7/5 mice). B: Remarkable inhibition of LTP by actinomycin in ACC neurons of CaMKIV transgenic (TG) mice (n = 6/4 mice). A-B: The insets showing averages of five EPSCs at baseline responses (pre) and at 25-30 min (30 min post) after the paired training (arrow); the dashed lines indicating the mean basal synaptic responses. C: Statistical results showing the significant inhibition of LTP by anisomycin at 15-20 and 25-30 min after induction of LTP in ACC neurons of wild-type mice. Summary results in control using data in Figure 1C. The dashed line indicating the mean basal synaptic responses. *P < 0.05 compared to control. D: Statistical results showing the significant inhibition of LTP by anisomycin at 0-5, 15-20 and 25-30 min after induction of LTP in ACC neurons of CaMKIV TG mice. Summary results in control using data in Figure 1D. The dashed line indicating the mean basal synaptic responses. **P < 0.01 compared to control. E: Anisomycin (Aniso) sensitive synaptic potentiation in ACC neurons of CaMKIV TG mice is larger than that of wild-type mice. F: Statistical results showing that Aniso sensitive synaptic potentiation in ACC neurons of CaMKIV TG mice is significantly larger than that of wild-type mice at 0-5, 15-20 and 25-30 min after induction of LTP. **P < 0.01 compared to wild-type mice.

Next, we investigated the effects of a translation inhibitor, anisomycin, on synaptic potentiation in ACC neurons induced by paired training. Anisomycin was used at a concentration of 20 μM, which inhibited the maintenance phase of hippocampal LTP [8,23]. In the ACC slices pretreated with 20 μM anisomycin, LTP induced by the paired training was significantly reduced in comparison with control (133.0 ± 10.5%, n = 8/5 mice, *P *< 0.05 compared with baseline at 25-30 min post-induction, Fig. 2A and 2C). Analysis of the time course of this effect in wild-type mice revealed that anisomycin significantly suppressed synaptic potentiation at 15-20 and 25-30 min but not at 0-5 min after LTP introduction (*P *< 0.05 compared with baseline, Fig. 2C), similar to the results obtained in the presence of actinomycin-D (Fig. 1C). This result also suggests that paired training itself can drive protein synthesis by activating translation at 15 min after LTP introduction in ACC neurons of wild-type mice.

In the CaMKIV transgenic mice, synaptic potentiation was also significantly reduced by anisomycin in comparison with control (151.6 ± 9.8% of baseline at 25-30 min post-induction, n = 6/4 mice, *P *< 0.05 compared with control; Fig. 2B and 2D). Analysis of the time course of this effect in CaMKIV transgenic mice revealed that anisomycin significantly suppressed synaptic potentiation at 0-5, 15-20 and 25-30 min after LTP introduction (*P *< 0.01 compared with baseline, Fig. 2D). This result also suggests that paired training can induce protein synthesis by activating translation immediately after LTP introduction in the ACC neurons of CaMKIV transgenic mice. By calculating the anisomycin sensitive synaptic potentiation in wild-type and CaMKIV transgenic mice, the effects of translation on synaptic potentiation caused by the paired training was compared between these two groups of mice (Fig. 2E). The anisomycin sensitive synaptic potentiation in the ACC neurons of the CaMKIV mice was significantly larger than that of the wild-type mice at 0-5, 15-20 and 25-30 min after introduction of LTP (*P *< 0.01 compared with baseline, Fig. 2F). These results indicate that not only gene transcription but also gene translation were markedly enhanced in the ACC neurons of CaMKIV transgenic mice compared to those of wild-type mice.

### Effects on AMPA and NMDA receptor mediated responses in ACC neurons

**Figure 3:**
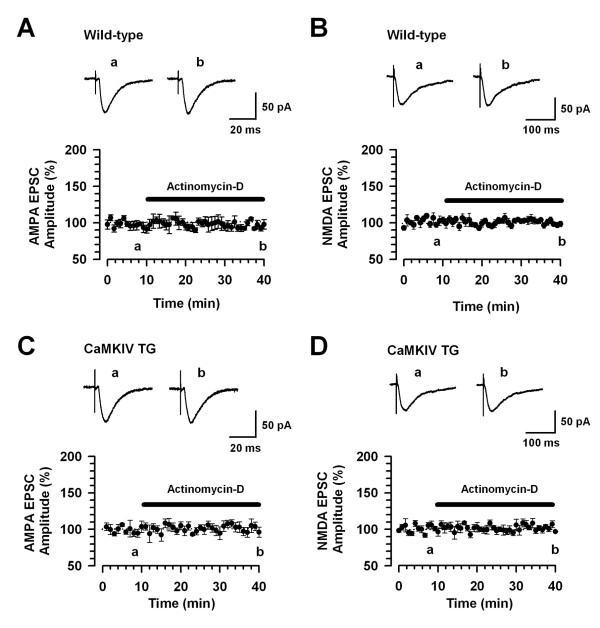
**A transcription inhibitor has no effect on basal synaptic transmission**. A: No effect of actinomycin-D (40 μM) on AMPA receptor-mediated EPSCs in ACC neurons of wild-type mice (n = 8/4 mice). B: No effect of actinomycin-D on NMDA receptor-mediated EPSCs in ACC neurons of wild-type mice (n = 6/3 mice). C: No effect of actinomycin-D on AMPA receptor-mediated EPSCs in ACC neurons of CaMKIV TG mice (n = 7/4 mice). D: No effect of actinomycin-D on NMDA receptor-mediated EPSCs in ACC neurons of CaMKIV TG mice (n = 6/3 mice). A-D: The dashed line indicating the mean basal synaptic responses. The insets showing averages of five EPSCs at baseline responses (a) and 25-30 min (b) after application of actinomycin-D (black bar).

To exclude the possible inhibition of basal synaptic transmission by translation and transcription inhibitors, we measured the α-amino-3-hydroxy-5-methyl-4-isoxazole-propionate (AMPA) or N-methyl-D-aspartic acid (NMDA) receptor-mediated basal EPSCs. In particular, the NMDA receptor is essential for the induction of LTP in forebrain areas including the ACC [4,24-26]. First, we investigated the effects of 40 μM actinomycin-D on AMPA receptor-mediated EPSCs. Actinomycin-D had almost no effect on the AMPA receptor-mediated EPSCs in the wild-type (n = 8/4 mice) and CaMKIV transgenic mice (n = 7/4 mice) (Fig. 3A and 3C, respectively). Next, we examined the effects of actinomycin-D on NMDA receptor-mediated EPSCs. Actinomycin-D also had no effect on NMDA receptor-mediated EPSCs in wild-type (n = 6/3 mice) and CaMKIV transgenic mice (n = 6/3 mice) (Fig. 3B and 3D, respectively).

**Figure 4:**
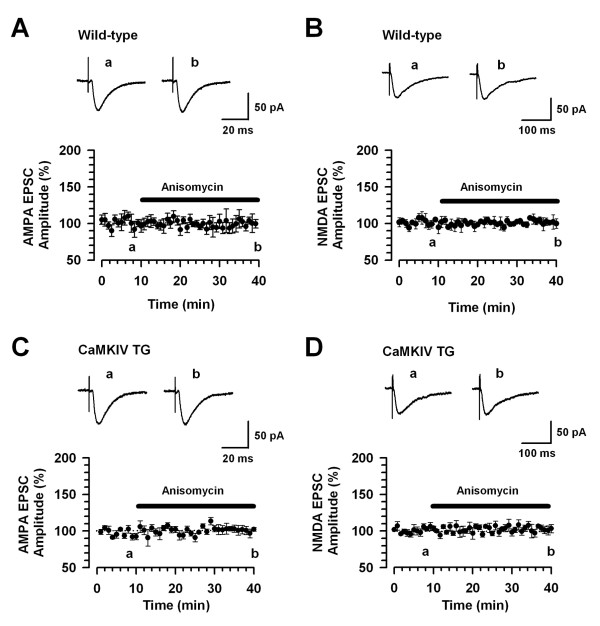
**A translation inhibitor has no effect on basal synaptic transmission**. A: No effect of anisomycin (20 μM) on AMPA receptor-mediated EPSCs in ACC neurons of wild-type mice (n = 8/5 mice). B: No effect of anisomycin on NMDA receptor-mediated EPSCs in ACC neurons of wild-type mice (n = 8/5 mice). C: No effect of anisomycin on AMPA receptor-mediated EPSCs in ACC neurons of CaMKIV transgenic (TG) mice (n = 8/5 mice). D: No effect of anisomycin on NMDA receptor-mediated EPSCs in ACC neurons of CaMKIV TG mice (n = 9/5 mice). A-D: The dashed line indicating the mean basal synaptic responses. The insets showing verages of five EPSCs at baseline responses (a) and 25-30 min (b) after application of anisomycin (black bar).

We further tested the effects of 20 μM anisomycin on AMPA receptor-mediated EPSCs. Similar to the effect of actinomycin-D, anisomycin had almost no effect on AMPA receptor-mediated EPSCs in wild-type (n = 8/5 mice) and CaMKIV transgenic mice (n = 8/5 mice) (Fig. 4A and 4C, respectively). We then examined the effects of anisomycin on NMDA receptor-mediated EPSCs. Anisomycin also showed no effect on NMDA receptor-mediated EPSCs in wild-type (n = 8/5 mice) and CaMKIV transgenic mice (n = 9/5 mice) (Fig. 4B and 4D, respectively). These results suggest that these inhibitors did not cause any functional modifications of AMPA and NMDA receptors.

### Expression of CaMKIV in ACC neurons of wild-type and CaMKIV transgenic mice

**Figure 5:**
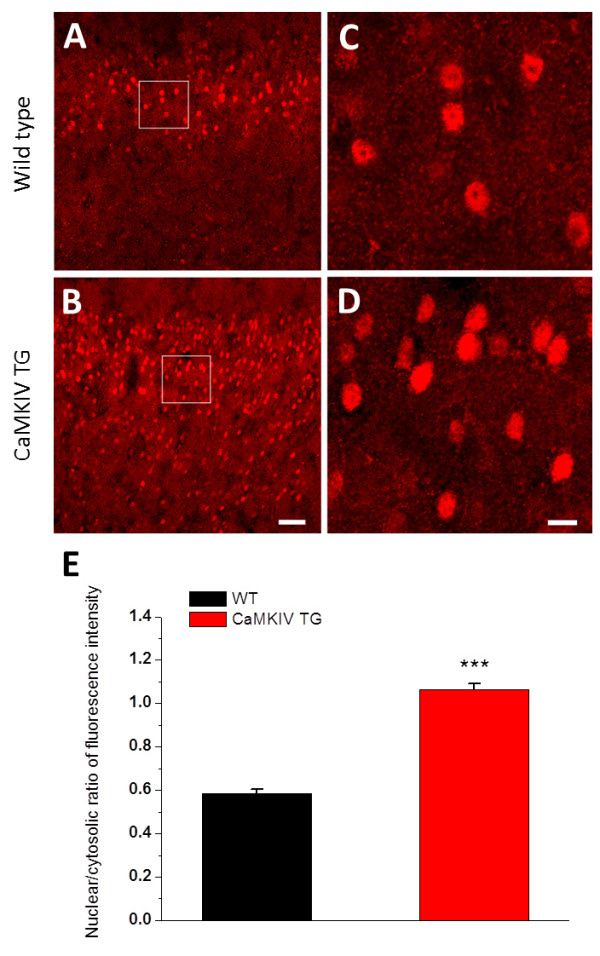
**Overexpression of CaMKIV in the ACC neurons of the CaMKIV transgenic mice**. A: CaMKIV immunoreactive (IR) neurons are mainly distributed in layer II/III of ACC in the wild-type (WT) mice. B: A larger number of CaMKIV-IR neurons are observed in layers II/III and V/VI of ACC in the CaMKIV transgenic (TG) mice. C-D: (C) and (D) showing enlarged images from rectangle areas in (A) and (B), respectively. Overexpression of CaMKIV immunoreactivity in the nuclei was observed in CaMKIV TG mice. E: Summary data of CaMKIV in ACC from the WT and CaMKIV TG mice. Bars equal to 50 μm in (A) and (B) and 10 μm in (C) and (D). ***, *P *< 0.001.

We performed immunostaining experiments to examine the expression of CaMKIV in the ACC neurons of wild-type and CaMKIV transgenic mice. We found that a larger number of CaMKIV immunopositive neurons were observed in the CaMKIV transgenic mice than in the wild-type mice in layers II/III and V/VI of ACC (Fig. 5A-D). We also tested the ratio of CaMKIV immunoreactivity between neuronal nuclei and cytoplasm in brain slices from wild-type and CaMKIV transgenic mice (Fig. 5E). An obvious increased expression of CaMKIV immunoreactivity in the nuclei was observed in CaMKIV transgenic mice (nuclei/cytosolic ratio of CaMKIV staining fluorescence intensity, 0.58 ± 0.02 in wild-type and 1.06 ± 0.03 in CaMKIV transgenic mice, *P *< 0.001).

### Expression of CaMKIV in cytosol and membrane compartments in the ACC neurons

**Figure 6:**
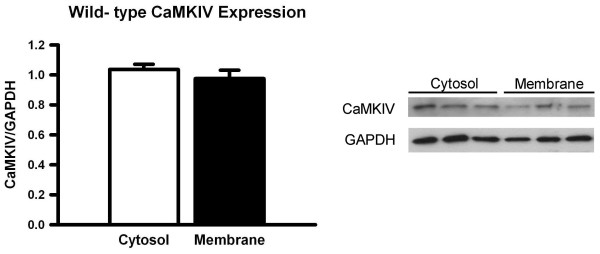
**Similar expression levels of CaMKIV in cytosol and membrane compartments of ACC neurons**. Western blot analysis showing CaMKIV expression in cytosol and membrane fractions. There is no difference in CaMKIV expression between cytosol and membrane samples.

In the present study, we found that synaptic potentiation caused by paired training was significantly suppressed by transcription and translation inhibitors in wild-type and CaMKIV transgenic mice (Fig. 1). Thus, we next examined the expression of CaMKIV within the cytosol and membrane compartments in the ACC neurons of wild-type mice by Western blot analysis. We observed expression of CaMKIV protein in both cytosol and membrane samples, and found no significant difference in the expression levels (Fig. 6). These results suggest that CaMKIV in membrane compartments may contribute to early synaptic potentiation in ACC neurons.

## Discussion

Activity-dependent activation of CaMKIV is critical for LTP induction in the ACC and is also critical for ACC-related brain functions such as trace fear memory [20]. Although CaMKIV is highly expressed in the ACC [19], little is known about how CaMKIV-induced protein synthesis contributes to the early-phase of synaptic potentiation in ACC neurons. The present study has demonstrated that synaptic potentiation in CaMKIV transgenic mice was remarkably inhibited by transcription and translation inhibitors in comparison with that in wild-type mice. Our biochemical and immunohistochemical studies revealed that CaMKIV was distributed in the membrane, cytosol and nucleus of ACC neurons. Especially, the nuclear/cytosolic ratio was significantly larger in the CaMKIV transgenic mice than in the wild-type mice, suggesting an enhanced expression of CaMKIV in the nuclei of CaMKIV transgenic mice. These results demonstrate for the first time that CaMKIV enhances short-term synaptic potentiation by activating protein synthesis in ACC neurons. Inhibitors of transcription and translation may inhibit the expression of short-lived proteins required for E-LTP, since these inhibitors were added 30 min before the induction of potentiation.

### Role of neuronal activity on protein synthesis-dependent LTP

LTP is initially achieved by posttranslational modifications of pre- and postsynaptic proteins following the induction of LTP and is later stabilized by transcription and translation [7,8]. Thus, LTP is divided into two phases such as early-phase and late-phase LTP; early-phase LTP depends on the activation of kinases and phosphatases, while late-phase LTP depends on new protein synthesis. Although it is generally believed that early-phase LTP is independent of protein synthesis, the role of protein synthesis in early-phase LTP in ACC neurons was poorly understood. Considering that late-phase LTP was disrupted by application of protein synthesis inhibitors before and during LTP induction while their application after LTP induction had no effect on its maintenance [9], the late-phase LTP would be necessary for a transient but not persistent upregulation of protein synthesis. Moreover, strong synaptic stimulation causes gene expression [27]. Thus, it is possible that the LTP induction stimulation itself can drive protein synthesis, thereby affecting the early-phase LTP. In this study, we found that the early synaptic potentiation induced by the paired training in ACC neurons of wild-type mice was significantly suppressed by protein synthesis inhibitors. Furthermore, Western blot and immunostaining analysis revealed that CaMKIV was indeed distributed in the membrane, cytosol and nucleus of ACC neurons. Accordingly, it is likely that the LTP induction stimuli readily recruit CaMKIV in membrane compartments, leading to protein synthesis. Taken together, these results suggest that the early-phase synaptic potentiation caused by the paired training in ACC neurons was partly mediated by a protein synthesis-dependent mechanism.

### Time course for transcription- and translation-related early synaptic potentiation

Although it is considered that early-phase LTP is independent of protein synthesis, a number of studies have demonstrated that early-phase LTP was partially or completely inhibited by protein synthesis inhibitors at CA1 [9,28,29], CA3 [10,30], and dentate gyrus [11] (see Table 1).

Similar to these reports, synaptic potentiation at 15-20 and 25-30 min after LTP induction was significantly suppressed by protein synthesis inhibitors in ACC neurons of wild-type mice, as shown in the present study. Considering that the synaptic potentiation at 0-5 min after LTP induction was not inhibited by protein synthesis inhibitors, protein synthesis-dependent synaptic potentiation begins at 15 min after LTP introduction in ACC neurons. This result indicates that blockade of transcription and translation selectively inhibited the early-phase LTP without affecting LTP induction.

It has been traditionally thought that new protein synthesis are performed in the cell body and transported to the activated synapses. However, numerous studies indicated that LTP induction stimuli induce local protein synthesis in dendritic spines of the postsynaptic neurons [31,32]. It is likely that new proteins are synthesized at the activated dendritic spines of ACC neurons. The time course of new protein synthesis in the postsynaptic neurons after LTP induction stimuli has been reported. In CA1 region of the hippocampus, protein kinase Mζ appeared 10 min after LTP induction [33]. In addition, it was also reported that LTP induction led to the increase of αCaMKII protein in the dendrites of stimulated hippocampal CA1 pyramidal neurons within 5 min of LTP induction [34]. Thus, protein synthesis in the postsynaptic neurons after LTP induction seems to be rapid, and such time course of protein synthesis is almost consistent with our data presented here. Together, these observations indicate that the mechanisms underlying early-phase LTP involve new protein synthesis.

### Role of CaMKIV in synaptic plasticity in ACC neurons

In the present study, we found that transcription- and translation-dependent synaptic potentiation was markedly enhanced in CaMKIV transgenic mice at 0-5, 15-20 and 25-30 min after induction of LTP. On the other hand, these inhibitors did not cause any effects on AMPA and NMDA receptor-mediated EPSCs in CaMKIV transgenic mice (Fig. 3 and 4), suggesting that the inhibition of synaptic potentiation by protein synthesis inhibitors was not due to a functional modification of AMPA and NMDA receptors. Thus, it is clearly indicated that the downstream targets of these receptors affected the early-phase LTP in ACC neurons of the CaMKIV transgenic mice. Several mechanisms could explain the role of CaMKIV in early-phase protein synthesis dependent LTP. First, synaptic proteins required for synaptic potentiation have been primed by overexpression of CaMKIV. Since our immunohistochemical study revealed that translocation of CaMKIV into the nuclei was markedly larger in the CaMKIV transgenic mice than in the wild-type mice, protein synthesis would be readily caused by LTP induction in the CaMKIV transgenic mice. Second, it has been demonstrated that CaMKIV phosphorylates and regulates the function of synaptic proteins including the actin binding protein synapsin I [35] and the microtubule regulator Stathmin [36]. Thus, the synaptic structural change in ACC neurons caused by CaMKIV may enhance the early-phase of synaptic potentiation.

The ACC is believed to be important in learning, memory and emotion, and pain in the mammalian brain [4,37-44], and LTP in the ACC is a vital key mechanism for cortical synaptic plasticity [6]. Through pharmacological and genetic studies, the molecular and cellular mechanisms of synaptic potentiation in the ACC are beginning to be elucidated [5,6]. The enhancement of neuronal activity caused by LTP inducing stimuli (strong tetanic stimulation, theta bust stimulation, paired training and spike-timing protocol) increases the release of the excitatory neurotransmitter glutamate in ACC synapses [5]. Subsequently, NMDA receptors including NR2A and NR2B can be activated, resulting in the elevation of intracellular Ca^2+ ^in postsynaptic cells [4]. The elevation of the intracellular Ca^2+ ^at postsynaptic cells activates a series of intracellular signaling pathways which contribute to LTP in the ACC. It has been well established that Ca^2+ ^binds to calmodulin (CaM), which activates CaM target proteins, such as Ca^2+^/CaM-dependent protein kinases (PKC, CaMKII and CaMKIV), CaM-activated ACs (AC1 and 8), and the CaM-activated phosphatase calcineurin in the hippocampus [45,46]. During synaptic potentiation within the ACC, we have reported that activation of AC1 and CaMKIV is important among the CaM target proteins mentioned above [19,25]. As the downstream target of AC1, cyclic-AMP (cAMP)-dependent protein kinase (PKA) is known to activate extracellular signal-regulated kinase (ERK) and ERK/MAPK (mitogen-activated protein kinase). Since ERK, c-Jun N-terminal kinase (JNK) and p38, family members of MAPKs, have been found to be critical in the induction of LTP in the ACC [47], AC1 may activate these MAPKs. Subsequently, the activated ERK/MAPK likely has multiple targets including CREB that is required for long-term synaptic changes in ACC neurons [19]. Although CaMIV is thought as the late signaling molecule, our observations clearly indicate that CaMKIV contributes to early enhancement of responses by promoting protein synthesis. Therefore, it is possible that CaMKIV is important synaptic plasticity in the ACC.

In summary, we demonstrate strong evidence that CaMKIV enhances synaptic plasticity in ACC neurons by promoting protein synthesis. Elucidating the molecular and cellular mechanisms in synaptic plasticity in the ACC may help us to determine the new insights of cortical plasticity and its related physiological and pathophysiological functions.

## Materials and methods

### Animals

All adult C57BL/6 mice were purchased from Charles River. Transgenic mice overexpressing CaMKIV were generated as previously described [48]. Briefly, we constructed a transgene that contained a α CaMKII promoter, a hybrid intron in the 5' untranslated leader, the coding region of CaMKIV fused with the Flag tag sequence at the N-terminus and a polyadenylation signal. The CaMKII promoter has been known to exhibit strong activity in regions of the forebrain including the hippocampus, cortex and striatum [49,50]. Among three lines of transgenic mice generated, transgenic line 2 showed the highest levels of transgene expression in the forebrain area [48]. Thus, transgene line 2 was used for the present experiments. Control wild-type mice were littermates of transgenic mice. All mice were maintained on a 12 h light: dark cycle with food and water provided ad libitum. The Animal Studies Committee at the University of Toronto approved all experimental protocols, which were in accordance with the guidelines of the Canadian Council on Animal Care.

### Slice preparation

Mice were anesthetized with 1-2% halothane and decapitated. Coronal brain slices (300 μm) containing the ACC from 6-8-week-old C57BL/6 male mice and mice with CaMKIV overexpression were prepared using standard methods [4,51]. Slices were transferred to a submerged recovery chamber with oxygenated (95% O_2 _and 5% CO_2_) artificial cerebrospinal fluid (ACSF) containing (in mM): NaCl, 124; KCl, 2.5; CaCl_2_, 2; MgSO_4_, 2; NaHCO_3_, 25; NaH_2_PO_4_, 1; glucose, 10; at room temperature for at least 1 h.

### Whole-cell patch-clamp recordings

All electrophysiological experiments were performed at room temperature. An Axioskop 2FS microscope (Zeiss, Germany) with infrared DIC optics was used for visualization of whole-cell patch-clamp recording. Excitatory postsynaptic currents (EPSCs) were recorded from layer II/III neurons with an Axon 200B amplifier (Molecular Devices, CA), and the stimulations were delivered by a bipolar tungsten stimulating electrode placed in layer V of the ACC slices. EPSCs were induced by repetitive stimulations (duration is 200 μs, intensity is adjusted to induce EPSCs with an amplitude of 50-100 pA) at 0.02 Hz and neurons were voltage-clamped at -70 mV. The recording pipettes (3-5 MΩ) were filled with solution containing (in mM): CsMeSO_3_, 102; TEA chloride, 5; NaCl, 3.7; EGTA, 0.2; HEPES, 20; MgATP, 2; NaGTP, 0.3; QX-314 chloride, 5 (adjusted to pH 7.2 with CsOH). LTP was induced with paired training within 12 min after establishing the whole-cell configuration to avoid washout of intracellular contents that are critical for the establishment of synaptic plasticity [4]. The induction protocol, referred to as paired training, for LTP involved pairing presynaptic 80 pulses at 2 Hz with postsynaptic depolarization at +30 mV. The N-methyl-d-aspartate (NMDA) receptor-mediated component of EPSCs was pharmacologically isolated in ACSF containing 6-cyano-2,3-dihydroxy-7-nitro-quinoxailne acid (CNQX; 25 μM). Neurons were voltage-clamped at -30 mV and NMDA receptor-mediated EPSCs were evoked at 0.05 Hz. Picrotoxin (100 μM) was always present to block γ-aminobutyric acid GABA_A _receptor-mediated inhibitory currents and monitored throughout the synaptic currents. Actinomycin-D and anisomycin (Sigma) were dissolved in DMSO, and diluted down to achieve a final concentration of 40 and 20 μM, respectively (in 0.01% DMSO). The ACC slices were pretreated with actinomycin-D or anisomycin for 30 min before LTP induction, which was maintained throughout the recording time. Control experiments using DMSO vehicle had no effects on baseline synaptic responses and LTP in ACC slices. Access resistance was 15-30 MΩ and was monitored throughout the experiment. Data were discarded if access resistance changed more than 15% during an experiment. Statistical comparisons were performed using the Student's t-test. Data were expressed as mean ± S.E.

### Immunohistochemistry

Wild-type and CaMKIV transgenic mice (n = 3 in each group) were anesthetized with isoflurane and perfused with 0.1 mol/L phosphate buffered saline (PBS, pH 7.2-7.4) via the ascending aorta followed by 4% paramaformaldehyde in 0.1 mol/L PB. The brains were then removed, and postfixed in the same fixative for 2 h before cryoprotection in PBS containing 30% sucrose overnight at 4°C. Every sixth sections of 25 μm thickness, serially cut through the brain in cryostat, were collected. Sections containing ACC were then used for CaMKIV immunoreactivity. Sections were sequentially incubated with the following solutions: (1) a solution of 3% bovine serum albumin (BSA, Sigma, St. Louis, USA), 0.3% Triton X-100 containing rabbit antibody against CaMKIV (ab3557; 1:1000, Abcam, USA) for 2 days at 4°C, (2) Rhodamine-conjugated goat anti-rabbit (1:200, Chemicon) in PBS containing 3% BSA and 0.3% Triton X-100 for 24 h at 4°C. All sections were rinsed with PBS (3 × 10 min) after each step. Sections were then mounted onto clean glass slides, airdried, coverslipped with a mixture of 50% (v/v) glycerin and 2.5% (w/v) triethylene diamine (anti-fading agent) in 0.01 M PBS, and observed with an laser-scanning confocal microscope (FV1000, Olympus, Japan). No staining was observed on brain sections when the primary antibody was omitted or replaced by normal rabbit serum from the protocol. Images were captured and analyzed with the assistance of Image-Pro Plus 5.0 software (Media Cybernetics, Inc., USA). Measurements of relative fluorescence intensity in the whole nuclear area as compared with that in the cytoplasm were applied [19]. All data was shown as a ratio between the nuclear and cytosolic area of each neuron. Only neurons with sharp boundaries and a well-defined nucleus were considered. Thirty neurons were measured from three different sections, for each animal, and averaged finally.

### Western blot analysis

Membrane preparation was performed as previously described [52] with minor changes. Briefly, ACC samples were dissected in cold D-PBS and resuspended in Buffer 1 (2 mM Tris-EDTA, 320 mM sucrose, 5 mM MgCl_2_, and 1× protease inhibitor cocktail, pH 7.4), and homogenized. Each sample was centrifuged at 1000xg for 10 min and the supernatants (S1) were recovered. The remaining pellet (P1) was then resuspended in Buffer 2 (50 mM Tris-HCl, 2 mM Tris-EDTA, 5 mM MgCl_2_, and 1× phosphatase inhibitor cocktail 1 and 2, pH 7.0) and centrifuged at 1000xg for 10 min, with its supernatant (S2) collected and combined with S1. The remaining pellet (P2) was resuspended in Buffer 2, and again centrifuged at 1000xg for 10 min., and its supernatant (S3) was combined with S1 and S2. Combined supernatant fractions (S1, S2 and S3) were finally centrifuged at 39,000xg for 30 min, the resulting supernatant contained the cytosolic fractions, and the resulting pellet (membrane fractions) was resuspended in Buffer 3 (50 mM Tris-HCl, 2 mM Tris-EDTA, 3 mM MgCl_2_, and 1× phosphatase inhibitor cocktail 1 and 2, pH 7.4). Western blot was performed as previously described [53]. Sample protein concentrations were quantified using Bradford assay, and electrophoresis of equal amounts of total protein was performed on NuPAGE 4-12% Bis-Tris Gels (Invitrogen, Carlsbad, CA). Separated proteins were transferred to polyvinylidene fluoride membranes (Pall Corporation, East Hills, NY) at 4°C overnight for analysis and were then probed with with primary CaMKIV antibody (1:1000, mouse monoclonal), followed by horseradish peroxidase (HRP)-coupled secondary antibody diluted at 1:3000 for 2 hours followed by enhanced chemiluminescence detection of the proteins with Western lightning chemiluminescence reagent plus (PerkinElmer Life Sciences). ImageJ software (National Institute of Health) was used to assess the density of immunoblots.

## List of abbreviations

ACC: anterior cingulate cortex; ACSF: artificial cerebrospinal fluid; AMPA: α-amino-3-hydroxy-5-methyl-4-isoxazolepropionic acid; CaMKIV: calcium/calmodulin-dependent protein kinase IV; CREB: cyclic AMP-responsive element binding protein; EPSC: excitatory postsynaptic current; LTP: long-term potentiation; NMDA: N-methyl D-aspartate receptor.

## Competing interests

The authors declare that they have no competing interests.

## Authors' contributions

HT is responsible for performance of electrophysiology and writing the manuscript. MGZ is responsible for performance of electrophysiology. VM, TC and GD are responsible for performance of biochemical and immunohistochemical studies. SK is responsible for experimental design. MZ is responsible for experimental design and writing the manuscript. All authors read and approved the final manuscript.
